# Differences in Outcome of Patients with Cardiogenic Shock Associated with In-Hospital or Out-of-Hospital Cardiac Arrest

**DOI:** 10.3390/jcm12052064

**Published:** 2023-03-06

**Authors:** Jonas Rusnak, Tobias Schupp, Kathrin Weidner, Marinela Ruka, Sascha Egner-Walter, Jan Forner, Thomas Bertsch, Maximilian Kittel, Kambis Mashayekhi, Péter Tajti, Mohamed Ayoub, Michael Behnes, Ibrahim Akin

**Affiliations:** 1 Department of Cardiology, Angiology, Haemostaseology and Medical Intensive Care, University Medical Centre Mannheim, Medical Faculty Mannheim, Heidelberg University, 68167 Mannheim, Germany; 2 European Center for AngioScience (ECAS), German Center for Cardiovascular Research (DZHK) Partner Site Heidelberg/Mannheim, 68167 Mannheim, Germany; 3 Institute of Clinical Chemistry, Laboratory Medicine and Transfusion Medicine, Nuremberg General Hospital, Paracelsus Medical University, 90419 Nuremberg, Germany; 4 Institute for Clinical Chemistry, Faculty of Medicine Mannheim, Heidelberg University, 68167 Mannheim, Germany; 5 Department of Internal Medicine and Cardiology, Mediclin Heart Centre Lahr, 77933 Lahr, Germany; 6 Gottsegen György National Cardiovascular Center, 1096 Budapest, Hungary; 7 Division of Cardiology and Angiology, Heart Center University of Bochum—Bad Oeynhausen, 32545 Bad Oeynhausen, Germany

**Keywords:** cardiogenic shock, prognosis, mortality, AMI, IHCA, OHCA

## Abstract

Cardiogenic Shock (CS) complicated by in-hospital (IHCA) or out-of-hospital cardiac arrest (OHCA) has a poor outcome. However, studies regarding the prognostic differences between IHCA and OHCA in CS are limited. In this prospective, observational study, consecutive patients with CS were included in a monocentric registry from June 2019 to May 2021. The prognostic impact of IHCA and OHCA on 30-day all-cause mortality was tested within the entire group and in the subgroups of patients with acute myocardial infarction (AMI) and coronary artery disease (CAD). Statistical analyses included univariable *t*-test, Spearman’s correlation, Kaplan–Meier analyses, as well as uni- and multivariable Cox regression analyses. A total of 151 patients with CS and cardiac arrest were included. IHCA on ICU admission was associated with higher 30-day all-cause mortality compared to OHCA in univariable COX regression and Kaplan–Meier analyses. However, this association was solely driven by patients with AMI (77% vs. 63%; log rank *p* = 0.023), whereas IHCA was not associated with 30-day all-cause mortality in non-AMI patients (65% vs. 66%; log rank *p* = 0.780). This finding was confirmed in multivariable COX regression, in which IHCA was solely associated with higher 30-day all-cause mortality in patients with AMI (HR = 2.477; 95% CI 1.258–4.879; *p* = 0.009), whereas no significant association could be seen in the non-AMI group and in the subgroups of patients with and CAD. CS patients with IHCA showed significantly higher all-cause mortality at 30 days compared to patients with OHCA. This finding was primarily driven by a significant increase in all-cause mortality at 30 days in CS patients with AMI and IHCA, whereas no difference could be seen when differentiated by CAD.

## 1. Introduction

Cardiac arrest in patients with cardiogenic shock (CS) is a feared complication that aggravates the severity of acute heart failure [1,2,3,4]. Survival rates of CS patients with in-hospital cardiac arrest (IHCA) or out-of-hospital cardiac arrest (OHCA) remain low despite improvement in treatment and implementation of extracorporeal life support (ECLS) [5,6].

In general, CS patients with cardiac arrest are a high-risk group. The vast majority of OHCAs and IHCAs is due to a cardiac reason, especially acute myocardial infarction (AMI) [7,8,9,10,11,12]. However, IHCA and OHCA are two diverse aetiologies with different outcomes, underlying causes for cardiac arrest, and patient characteristics [13]. Furthermore, comorbidities are more prevalent in patients with IHCA [13]. Survival rates range from 15% to 34% for IHCA and from 0% to 18% for OHCA, with an average of 8% [14].

In a small observational study of 43 patients, treated with conventional cardiopulmonary resuscitation (CPR) or extracorporeal CPR after IHCA due to AMI, higher survival rates to discharge could be seen in patients with ST-segment elevation myocardial infarction (STEMI), initial shockable rhythm and a low time to coronary intervention [15]. Current European Resuscitation Council (ERC) guidelines recommend coronary angiography in adult patients with return of spontaneous circulation (ROSC) after cardiac arrest due to a STEMI [14]. Moreover, it is stated that emergent cardiac catheterization laboratory evaluation should be considered in patients with ROSC after OHCA without ST-elevation on the ECG if there is a high probability for total occlusion of a coronary artery. The large randomized controlled TOMAHAWK trial showed that an immediate was not superior to a delayed coronary angiography in patients with OHCA and suspected cardiac cause [16]. However, in the ERC guidelines, no specific recommendation is given for patients with IHCA and a high probability of cardiac occlusion.

Therefore, a more distinct investigation for patients with IHCA caused by CS is needed. Patients with IHCA compared to OHCA have different underlying pathologies, and therefore, distinct management appears in the ongoing workup. The purpose of this study is to compare patients with cardiac arrest and underlying CS, as well as the prognostic differences in the outcomes of these two groups throughout the overall cohort and the subset of patients with or without AMI and known coronary artery disease (CAD). Furthermore, the present study gives insights into the possible underlying cause of the differences in outcomes.

## 2. Materials and Methods

### 2.1. Study Patients, Design, and Data Collection

The present study prospectively included all consecutive patients presenting with CS on ICU admission to the internal intensive care unit (ICU) at the University Medical Center Mannheim, Germany, from June 2019 to May 2021. All relevant clinical data related to the index event was documented using the electronic hospital information system as well as the IntelliSpace Critical Care, and anesthesia information system (ICCA, Philips, Philips GmbH Market DACH, Hamburg, Germany) implemented on the ICU, organizing patient data, including admission documents, vital signs, laboratory values, treatment data and consult notes.

The presence of CS, as well as important laboratory data, ICU-related scores, hemodynamic measurements, and ventilation parameters, was assessed on the day of ICU admission as well as on days 2, 3, 4, 7, and 8 thereafter, respectively.

Further data being documented contained baseline characteristics, prior medical history, length of index hospital stay, data derived from imaging diagnostics, as well as pharmacological therapies and resuscitation status. Documentation of source data was performed by intensivists and ICU nurses during routine clinical care.

The present study derived from an analysis of the “Cardiogenic Shock Registry Mannheim” (CARESMA-registry), representing a prospective single-center registry including consecutive patients presenting with CS being acutely admitted to the ICU for internal medicine of the University Medical Center Mannheim (UMM), Germany (clinicaltrials.gov identifier: NCT05575856). The registry was carried out according to the principles of the Declaration of Helsinki and was approved by the medical ethics committee II of the Medical Faculty Mannheim, University of Heidelberg, Germany.

### 2.2. Inclusion and Exclusion Criteria, Study Endpoints

All consecutive patients admitted to the ICU were screened for CS as the underlying diagnosis for ICU admission for the purpose of the current investigation. Additionally, for the study, only patients with CS and a recorded cardiac arrest that had been succesfully aborted or was still ongoing at the time of ICU admission were included. Patients without documented cardiac arrest as ICU admission diagnosis or patients with cardiac arrest and aborted cardiopulmonary resuscitation before admission to the ICU were not included in the present study.

Risk stratification was performed according to the site of cardiac arrest (IHCA vs. OHCA). The IHCA group consisted of patients with cardiac arrest occurring inside the hospital. In detail, this included patients treated in the emergency department, outpatient clinic, and general ward, as well as patients in the diagnostic area, such as coronary angiography and computed tomography. The study did not include patients with CS who had already been hospitalized in the ICU and experienced a cardiac arrest the day after ICU admission. Four patients in the IHCA group had coronary angiography before cardiac arrest. The OHCA group consisted of patients with a cardiac arrest that occurred outside of the hospital. Patients dying before admitting ICU or catheterization laboratory were excluded from the study. However, patients with OHCA and ongoing cardiopulmonary resuscitation on ICU admission or appearance in the catheterization laboratory were included when CS was the underlying cause of OHCA. No further exclusion criteria were applied.

Diagnosis of CS was determined according to the current recommendations of the Acute Cardiovascular Care Association of the European Society of Cardiology [17]. Accordingly, CS was defined by hypotension (SBP < 90 mmHg) for more than 30 min despite adequate filling status or the need for vasopressor or inotropic therapy to achieve SBP > 90 mmHg. Additionally, signs of end-organ hypoperfusion must be present such as oliguria with urine output <30 mL/hour, altered mental status, cold, clammy skin, and increased lactate >2 mmol/L.

During the ICU treatment, different circulatory and oxygenation goals were aimed. Therefore, all patients treated for cardiac arrest had a mean arterial pressure (MAP) target of 65 mmHg and should have a partial oxygen pressure (PaO_2_) of 65 mmHg. After admission, parameters of mechanical ventilation and circulatory support were increased to reach these goals and lowered if the values of MAP and PaO_2_ were too high.

The subgroup of AMI included patients with STEMI and non–ST-segment elevation myocardial infarction (NSTEMI). According to current European guidelines, AMI was defined as the presence of an acute myocardial injury with clinical evidence of acute myocardial ischemia prior to cardiac arrest [18,19]. Furthermore, the definition was based on the detection of a rise or fall of cardiac troponine values, with at least one value above the 99th percentile upper reference limit. At least one of the following conditions must exist: symptoms of myocardial ischemia prior to cardiac arrest, new ischemic ECG changes, development of pathological Q waves, imaging evidence of new loss of viable myocardium or new regional wall motion abnormality in a pattern consistent with an ischemic etiology, or identification of a coronary thrombus by angiography. The results of coronary angiography at index hospitalization were retrieved to update the CAD diagnosis in these patients [18,20].

All-cause mortality at 30 days was documented using the electronic hospital information system and by directly contacting state resident registration offices (‘bureau of mortality statistics’). Identification of patients was verified by place of name, surname, day of birth, and registered living address. No patient was lost to follow-up with regard to all-cause mortality at 30 days. The beginning of the follow-up period was the day of ICU admission, which was the day the patient was transferred to the ICU. Therefore, in IHCA patients, the day of admission to the hospital might not be the day of ICU admission. The period of 30 days was chosen to investigate the impact of CS and its consequences in the acute setting, and a longer follow-up period would have led to more influence of confounding parameters on all-cause mortality.

### 2.3. Statistical Methods

Quantitative data is presented as mean ± standard error of the mean (SEM), median and interquartile range (IQR), and ranges depending on the distribution of the data. They were compared using the Student’s *t*-test for normally distributed data or the Mann–Whitney U test for nonparametric data. Deviations from a Gaussian distribution were tested by the Kolmogorov–Smirnov test. Qualitative data is presented as absolute and relative frequencies and were compared using the chi-square test or Fisher’s exact test, as appropriate.

Kaplan–Meier analyses according to IHCA and OHCA on ICU admission were performed within the entire cohort as well as in the groups of non-AMI and AMI as well as non-CAD and CAD. Univariable hazard ratios (HR) were given together with 95% confidence intervals. Thereafter, multivariable Cox regression models were developed using the “forward selection” option, where only statistically significant variables (*p* < 0.05) were included and analyzed simultaneously.

The results of all statistical tests were considered significant for *p* ≤ 0.05. SPSS (Version 25, IBM, Armonk, New York, NY, USA) and GraphPad Prism (Version 9, GraphPad Software, San Diego, CA, USA) were used for statistics.

## 3. Results

**Figure 1:**
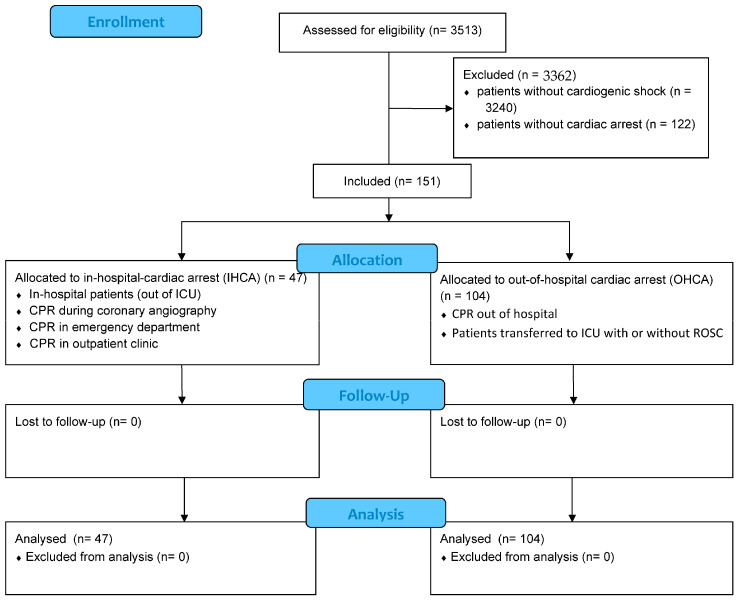
Flow chart of inclusion process of patients with cardiogenic shock (CS).

As illustrated in Figure 1, the final entire cohort comprised 151 patients with CS and aborted cardiac arrest with a median age of 69 years. As seen in Table 1, patients with IHCA were less often males (48.9% vs. 72.1%; *p* = 0.006) and showed a higher body temperature (36.1 °C vs. 35.2 °C; *p* = 0.003) as well as lower systolic blood pressure (100 mmHg vs. 114 mmHg; *p* = 0.002). However, patients with IHCA suffered more often from diabetes mellitus (46.8% vs. 28.2%; *p* = 0.025), congestive heart failure (38.3% vs. 18.3%; *p* = 0.008), chronic kidney disease (42.6% vs. 16.3%; *p* = 0.001) and stroke (21.3% vs. 4.8%; *p* = 0.002). Medication on ICU admission differed not significantly between the groups.

Due to the study design, all patients had a shock stage E (Table 2). The cause of CS was evenly distributed between the groups, and no differences could be seen in transthoracic echocardiographic parameters. Regarding laboratory parameters, lower values of D-dimer (3.16 mg/L vs. 24.18 mg/L; *p* = 0.001) were seen in patients with IHCA, whereas bilirubin (0.78 mg/dL vs. 0.61 mg/dL; *p* = 0.027) and CRP (21 mg/L vs. 4 mg/L; *p* = 0.001) were lower in patients with OHCA. Time in ICU was shorter in patients with IHCA (2 days vs. 6 days; *p* = 0.001).

Furthermore, patients with IHCA showed more often shockable rhythms (71.7% vs. 38.8%; *p* = 0.001), had a significantly shorter time to ROSC (10 min vs. 15 min; *p* = 0.017), and were less often treated with targeted temperature management (TTM; 12.8% vs. 60.6%; *p* = 0.001) compared to patients with OHCA (Table 3). Moreover, CS patients with IHCA needed less frequent mechanical ventilation (67.4% vs. 96.1%; *p* = 0.001) with a shorter duration of mechanical ventilation (2 days vs. 5 days; *p* = 0.001) and lower PaCO2 (39 mmHg vs. 46 mmHg, *p* = 0.018) as well as shorter duration of continuous renal replacement therapy (CRRT; 16 h vs. 70 h; *p* = 0.008). Regarding coronary status, patients with IHCA had a significantly longer time to coronary angiography (262 min vs. 121 min; *p* = 0.029).

**Figure 2:**
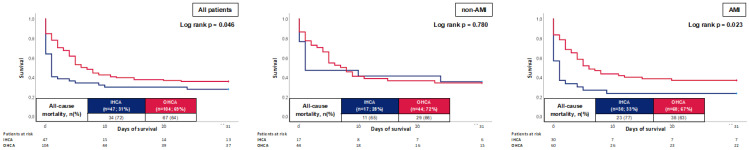
Prognostic impact of the OHCA and IHCA on all-cause mortality at 30 days within the entire cohort (**left panel**), as well as in the cohort of non-AMI patients (**middle panel**) and AMI (**right panel**).

At 30 days, the primary endpoint of all-cause mortality occurred in 72.3% of patients with IHCA and 64.4% of patients with OHCA. Accordingly, the risk of all-cause mortality was higher in patients with IHCA (log rank *p* = 0.046; HR = 0.557; 95% CI 0.327–0.949; *p* = 0.031) in Kaplan–Meier analyses as well as in multivariable Cox regression (Figure 2, left panel and Table 4). However, this finding could solely be verified in the group of patients with AMI, in which patients with IHCA showed significantly higher mortality compared to patients with OHCA (77% vs. 63%; log rank *p* = 0.023), whereas no difference could be seen in patients without AMI (65% vs. 66%; log rank *p* = 0.780) (Figure 2, middle and right panel).

**Figure 3:**
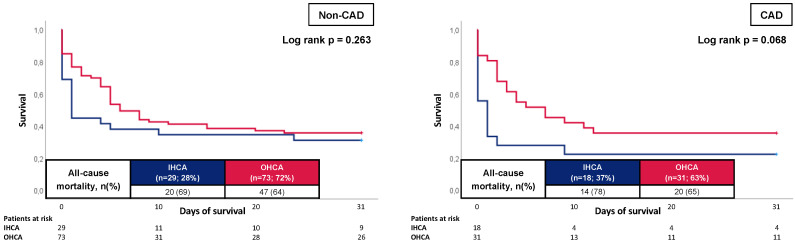
Prognostic impact of the OHCA and IHCA on all-cause mortality at 30 days within in the cohort of non-CAD patients (**left panel**) and CAD patients (**right panel**).

Kaplan–Meier analysis was performed in the subgroup of patients with and without CAD (Figure 3). Here, solely in the subgroup of CAD patients, a tendency toward a worse outcome for individuals with IHCA could be noticed (78% vs. 65%; log rank *p* = 0.068). In the subgroup of patients without CAD, no difference could be demonstrated between patients with IHCA and OHCA (69% vs. 64%; log rank *p* = 0.263).

**Figure 4:**
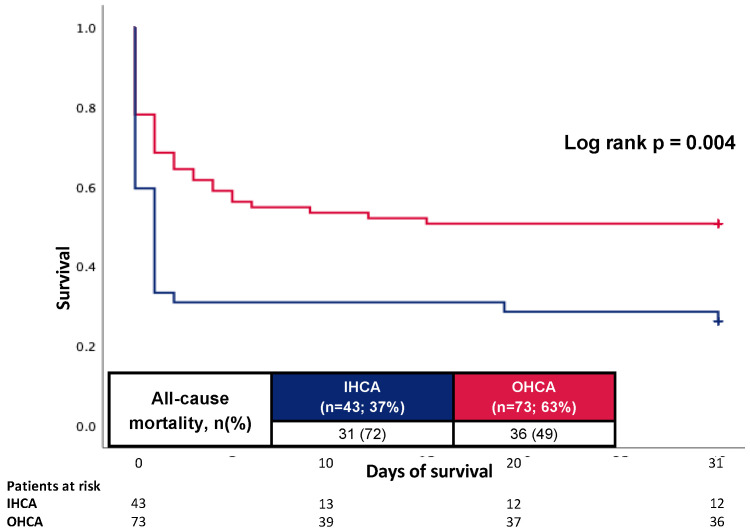
Prognostic impact of the OHCA and IHCA on all-cause mortality at 30 days in the cohort without patients died due to hypoxic-ischemic encephalopathy and limitations of care.

As illustrated in Figure 4, even after excluding patients with death due to hypoxic-ischemic encephalopathy and limitation of care, patients with IHCA showed an increased 30-day all-cause mortality (72% vs. 49%; log rank *p*-value = 0.004).

After multivariable adjustment, IHCA was associated with reduced risk of all-cause mortality at 30 days in patients with AMI (HR = 2.477; 95% CI 1.258—4.879; *p* = 0.009), whereas no difference could be seen in patients without AMI (Table 5). In this Cox regression model, WBC (HR 1.100; 95% CI 1.023—1.183; *p* = 0.010) and platelets (HR 0.991; 95% CI 0.985–0.998; *p* = 0.014) showed significant association with the primary endpoint of all-cause mortality at 30 days in the non-AMI group. In the AMI group, solely IHCA was associated with the primary endpoint.

## 4. Discussion

The present study investigates the prognostic differences in patients admitted to ICU with CS complicated by IHCA or OHCA. The data provides new insights regarding the outcome in these specific patient groups. First, CS patients with cardiac arrest have a high mortality rate irrespective of the in-hospital or out-of-hospital origin. Second, CS patients with IHCA showed a worse outcome compared to CS patients with OHCA. This finding is driven by increased mortality of CS patients with IHCA in the setting of AMI. Interestingly, higher mortality rates occurred, irrespective of the fact that compared to OHCA, more shockable rhythms were the underlying cause of IHCA. No significant difference could be seen in the subgroup of patients with known CAD.

In total, 30-day all-cause mortality rates were higher in CS patients with IHCA compared to OHCA, which is in contrast to other studies investigating cardiac arrest patients of any cause [13,21,22,23]. This might be due to the study design, which included solely CS patients with IHCA occurring in the general ward, in the emergency department, or outpatient clinic, whereas IHCAs in ICU were not included in the study. Missing monitoring in the general ward and in the outpatient clinic is a risk factor for worse outcomes in IHCA [24,25]. Furthermore, patients with OHCA that did not survive until ICU admission were excluded from the study. This might explain the lower mortality rate in CS patients with OHCA. Moreover, cardiac arrest is known to have a greater influence on in-hospital mortality in patients with lower stages of CS, as demonstrated in a retrospective analysis of Jentzer et al. in which 9898 patients with CS were included [26]. Therefore, the specific cohort of the present study solely consisted of patients with shock stage E, in which IHCA or OHCA might have little influence on the outcome. However, even after multivariable Cox regression, IHCA showed a negative impact in the group of patients with AMI, which shows that there might be a specific difference between IHCA and OHCA in the presence of AMI.

AMI or myocardial ischemia without an infarction are common causes in patients with IHCA [9,10,11]. In a retrospective single-center analysis of 1041 patients with IHCA, the leading cause of cardiac arrest was an AMI. [10] In a study by Tirkkonen et al., half of the patients with IHCA had an underlying cardiac cause for cardiac arrest and showed a shockable rhythm in 13% of the cases. [9] In the present study, patients with IHCA had a shockable rhythm in nearly 72%, which is due to the specific cohort of patients with CS. However, mortality rates were still higher in patients with IHCA compared to OHCA, irrespective of the prognostic beneficial shockable rhythm. This occurs to be an unusual outcome at first sight. However, a retrospective analysis of propensity score-matched cohorts of patients presenting with VT/VF showed that survivors of IHCA were associated with increased risk for all-cause mortality at 2.5 years compared to OHCA survivors [27]. This finding is in line with the present study, and the higher mortality rates in both registries might be explained by the higher rate of comorbidities such as diabetes mellitus and prior heart failure. However, it shows that the cohort of patients with CS complicated by IHCA is a common and specific high-risk category of patients who are in need of special attention. Moreover, in a meta-analysis and systemic review of 27,102 patients with IHCA, it could be shown that parts of the classic “H’s and T’s” of advanced cardiac life support do appear less often in the setting of IHCA indicating that other diagnostic and treatment strategies might be beneficial [11].

In the present study, patients with OHCA were more often treated with TTM, which may have had an impact on the outcome as it might reduce myocardial reperfusion injury and infarct size after myocardial ischemia [28,29,30]. In in vivo models, hypothermia was able to reduce infarct size as well as post-ischemic contractile and mitochondrial dysfunction [28,31]. Furthermore, hypothermia reduces the release of reactive oxygen species during ischemia and upon reperfusion in different rat and rabbit models [31,32]. Therefore, the higher rate of TTM could have an impact on the better outcome of patients with OHCA.

Moreover, a more severe underlying CAD might explain the higher mortality rates of patients with IHCA, who were more likely to suffer from diabetes mellitus and stroke. Especially diabetes mellitus is a known risk factor for CAD and is able to aggravate this disease. After differentiation for known CAD, no significant difference could be seen regarding the primary endpoint. Therefore, a known CAD might not be a reliable, independent indicator to perform primary PCI after IHCA. However, in the present study, CS patients with IHCA showed a slightly higher rate of culprit lesions in coronary angiography, indicating that these patients might profit from an earlier coronary angiography when a coronary ischemic event is suspected. Furthermore, time to coronary angiography is a known relevant risk predictor in patients with IHCA, which was significantly higher in patients with IHCA compared to patients with OHCA [15]. In summary, the results from the present study indicate that there should be a higher awareness of ischemic cardiac events in the setting of CS complicated by IHCA, regardless of a present CAD. Current ERC guidelines do not explicitly recommend emergent cardiac catheterization laboratory evaluation in patients with ROSC after IHCA without ST-elevation on the ECG if there is a high probability for total occlusion of a coronary artery [14].

This study has several limitations. Due to the single-center and observational study design, results may be influenced by measured and unmeasured confounding, although adjustments for potential cofounders were performed using multivariable Cox regression. Furthermore, there are regional differences in the treatment and care of patients with IHCA and OHCA, including different team compositions and expertise. Therefore, the results of the study cannot be transferred to other medical centers in general, and large randomized controlled trials (RCT), as well as multicenter studies investigating the different outcomes of patients with OHCA and IHCA associated with CS, are needed to address these differences. In the present study, patients dying before ICU admission were not included, and time to hospitalization for patients with OHCA might have influenced the results. Since patients might have been transferred to a rehabilitation facility after overcoming the critical phase, data from these institutions has not necessarily been sent to our institution. Furthermore, at the end of the index hospital stay, no standardized neurological outcome score was indicated in patients’ reports. Taking this together, no details on a neurological outcome, such as CPC score, can be given as this was beyond the scope of the study. Furthermore, this study was carried out during the COVID-19 pandemic. Therefore, admissions with CS and cardiac arrest were lower than expected, and the sample size was smaller than calculated. The results of the present study must therefore be interpreted with caution. However, care of critically ill patients was accomplished all time. Finally, the effects of IHCA and OHCA on long-term outcomes were beyond the scope of the present study.

The current study gives new insights into the different outcomes of patients with IHCA and underlying CS compared to patients with OHCA and CS. From these results, no direct conclusion can be made regarding the clinical practice and management of these patients. However, it shows that patients with IHCA and CS are a specific group that needs further investigation in larger and multicentric studies to specify treatment and workup of patients with IHCA and suspected underlying cardiac cause, especially in the setting of AMI.

## 5. Conclusions

In conclusion, the present study gives new insights into the specific patient group suffering from CS and cardiac arrest, especially in the setting of AMI. The data showed that patients with IHCA due to CS had a worse outcome compared to CS patients with OHCA, even after adjustment in multivariate Cox regression and in Kaplan–Meier analysis. Especially CS patients with IHCA showed a higher mortality rate when AMI was the underlying cause, which might be due to a longer time for coronary angiography. These findings suggest that patients with CS and IHCA caused by AMI are a specific group of patients that are in need of further investigation and special attention.

## Figures and Tables

**Table 1:** Baseline characteristics of CS patients with IHCA and OHCA.

	All Patients (n = 151)		IHCA (n = 47)		OHCA (n = 104)		*p*-Value
** Age**, median; (IQR)	69	(58–78)	74	(60–78)	67	(58–78)	0.101
** Male sex**, n (%)	98	(64.9)	23	(48.9)	75	(72.1)	**0.006**
** Body mass index**, kg/m^2^ (median, (IQR))	26.65	(24.20–30.40)	27.10	(24.20–31.13)	26.25	(24.23–29.88)	0.530
** Physiologic parameters** (median, (IQR))							
Body temperature (°C)	35.4	(34.3–36.3)	36.1	(35.0–36.9)	35.2	(34.0–36.2)	**0.003**
Heart rate (bpm)	86	(72–108)	92	(80–112)	84	(71–106)	0.149
Systolic blood pressure (mmHg)	110	(95–131)	100	(81–120)	114	(98–136)	**0.002**
Respiratory rate (breaths/min)	19	(16–22)	19	(17–24)	19	(16–22)	0.686
** Cardiovascular risk factors**, n (%)							
Arterial hypertension	97	(64.2)	32	(68.1)	65	(62.5)	0.507
Diabetes mellitus	51	(34.0)	22	(46.8)	29	(28.2)	**0.025**
Hyperlipidemia	65	(43.0)	24	(51.1)	41	(39.4)	0.181
Smoking	50	(33.1)	19	(40.4)	31	(29.8)	0.199
** Prior medical history**, n (%)							
Coronary artery disease	49	(32.5)	18	(28.3)	31	(29.8)	0.683
Congestive heart failure	37	(24.5)	18	(38.3)	19	(18.3)	**0.008**
Atrial fibrillation	33	(21.9)	12	(25.5)	21	(20.2)	0.462
Chronic kidney disease	37	(24.5)	20	(42.6)	17	(16.3)	**0.001**
Stroke	15	(9.9)	10	(21.3)	5	(4.8)	**0.002**
COPD	27	(17.9)	8	(17)	19	(18.3)	0.853
Liver cirrhosis	3	(2.0)	1	(2.1)	2	(1.9)	0.934
** Medication on admission**, n (%)							
ACE-inhibitor	43	(33.1)	14	(33.3)	29	(33.0)	0.966
ARB	21	(16.0)	8	(19.0)	13	(14.6)	0.518
Beta blocker	57	(43.8)	23	(54.8)	34	(38.6)	0.083
ARNI	3	(2.3)	1	(2.4)	2	(2.2)	0.962
Aldosterone antagonist	18	(13.8)	4	(9.5)	14	(15.9)	0.324
Diuretics	48	(36.4)	20	(47.6)	28	(31.1)	0.066
ASA	32	(21.2)	13	(27.7)	19	(18.3)	0.191
P2Y12 inhibitor	11	(7.3)	4	(8.5)	7	(6.7)	0.697
Statin	51	(38.9)	19	(45.2)	32	(36.0)	0.309

ACE, angiotensin-converting enzyme; ARB, angiotensin receptor blockers; ARNI, angiotensin receptor neprilysin inhibitor; ASA, acetylsalicylic acid; COPD, chronic obstructive pulmonary disease; IHCA, in-hospital cardiac arrest; IQR, interquartile range; OHCA, out-of-hospital cardiac arrest. Level of significance *p* < 0.05.

**Table 2:** Shock-related data, follow-up data, and endpoints.

	All Patients (n = 151)		IHCA (n = 47)		OHCA (n = 104)		*p*-Value
** Cause of CS**, n (%)							
Acute myocardial infarction	90	(59.6)	30	(63.8)	60	(57.7)	
Arrhythmic	16	(10.6)	4	(8.5)	12	(11.5)	
ADHF	25	(16.6)	8	(17.0)	17	(16.3)	
Pulmonary embolism	11	(7.3)	2	(4.3)	9	(8.7)	0.905
Vitium	2	(1.3)	1	(2.1)	1	(1.0)	
Cardiomyopathy	6	(4.0)	2	(4.3)	4	(3.8)	
Aortic dissection	1	(0.7)	0	(0.0)	1	(1.0)	
** Classification of CS**, n (%)							
Stage A	0	(0.0)	0	(0.0)	0	(0.0)	-
Stage B	0	(0.0)	0	(0.0)	0	(0.0)	
Stage C	0	(0.0)	0	(0.0)	0	(0.0)	
Stage D	0	(0.0)	0	(0.0)	0	(0.0)	
Stage E	151	(100.0)	47	(100.0)	104	(100.0)	
** Transthoracic echocardiography**							
LVEF >55%, (n, %)	15	(9.9)	7	(14.9)	8	(7.7)	
LVEF 54–41%, (n, %)	12	(7.9)	1	(2.1)	11	(10.6)	
LVEF 40–30%, (n, %)	34	(22.5)	9	(19.1)	25	(24.0)	0.242
LVEF <30%, (n, %)	72	(47.7)	25	(53.2)	47	(45.2)	
LVEF not documented, (n, %)	18	(11.9)	5	(10.6)	13	(12.5)	
VCI, cm (median, (IQR))	1.8	(1.5–2.2)	2.0	(1.8–2.3)	1.8	(1.5–2.2)	0.248
TAPSE, mm (median, (IQR))	17	(14–23)	19	(13–23)	17	(14–21)	0.754
** Baseline laboratory values** (median, (IQR))							
pH	7.26	(7.15–7.34)	7.29	(7.16–7.36)	7.25	(7.15–7.34)	0.400
Lactate (mmol/L)	4.0	(2.2–9.4)	4.4	(1.6–10.4)	3.8	(2.2–8.8)	0.871
Sodium (mmol/L)	139	(136–141)	139	(135–143)	139	(136–140)	0.684
Potassium (mmol/L)	4.2	(3.6–4.6)	4.2	(3.8–4.7)	4.2	(3.5–4.6)	0.595
Creatinine (mg/dL)	1.44	(1.11–1.86)	1.49	(1.03–2.48)	1.41	(1.18–1.76)	0.471
Hemoglobin (g/dL)	13	(11.2–14.3)	12.1	(10.3–13.8)	13.2	(11.5–14.4)	0.206
WBC (10^6^/mL)	16.19	(12.22–20.58)	15.56	(12.19–19.66)	16.39	(12.31–21.81)	0.475
Platelets (10^6^/mL)	227	(176–274)	231	(179–265)	225	(175–276)	0.830
INR	1.18	(1.08–1.41)	1.31	(1.08–1.58)	1.17	(1.08–1.35)	0.151
D-dimer (mg/L)	19.17	(8.55–32.00)	3.16	(1.15–10.50)	24.18	(14.28–32.00)	**0.001**
AST (U/L)	167	(79–478)	109	(40–770)	167	(110–416)	0.742
ALT (U/L)	111	(58–233)	85	(29–362)	115	(71–202)	0.530
Bilirubin (mg/dL)	0.63	(0.45–0.92)	0.78	(0.54–1.17)	0.61	(0.41–0.79)	**0.027**
Troponin I (µg/L)	0.895	(0.191–5.912)	1.470	(0.178–20.709)	0.731	(0.201–5.047)	0.333
NT-pro BNP (pg/mL)	1122	(328–8695)	5486	(166–15834)	1047	(339–4462)	0.186
Procalcitonin (ng/mL)	0.26	(0.07–1.05)	0.33	(0.10–1.23)	0.23	(0.06–1.22)	0.789
CRP (mg/L)	5	(4–27)	21	(5–58)	4	(4–19)	**0.001**
**Primary endpoint**							
All-cause mortality at 30 days, n (%)	101	(66.9)	34	(72.3)	67	(64.4)	0.338
Cause of death, n (%)							
- Cardiogenic shock	66	(43.7)	29	(61.7)	37	(35.6)	1.000
- Hypoxic-ischemic encephalopathy	21	(13.9)	3	(6.4)	18	(17.3)	
- Limitation of care	6	(4.0)	0	(0.0)	6	(5.8)	
- Other cause	9	(5.9)	2	(4.3)	7	(6.7)	
** Follow-up data**, n (%)							
ICU time, days (median, (IQR))	4	(2–9)	2	(1–6)	6	(2–10)	**0.001**
Death ICU, n (%)	100	(66.2)	33	(70.2)	67	(64.4)	0.486

ADHF, acute decompensated heart failure; ALT, alanine aminotransferase; AST, aspartate aminotransferase; CRP, C-reactive protein; CS, cardiogenic shock; DIC, disseminated intravascular coagulation; GFR, glomerular filtration rate; ICU, intensive care unit; IHCA, in-hospital cardiac arrest; INR, international normalized ratio; IQR, interquartile range; LVEF, left ventricular ejection fraction; NT-pro BNP, N-terminal pro-B-type natriuretic peptide; OHCA, out-of-hospital cardiac arrest; TAPSE, tricuspid annular plane systolic excursion; VCI, vena cava inferior; WBC, white blood cells. Level of significance *p* < 0.05. Bold type indicates statistical significance.

**Table 3:** Data regarding cardiac arrest and mechanical organ support.

	All Patients (n = 151)		IHCA (n = 47)		OHCA (n = 104)		*p*-Value
** Cardiopulmonary resuscitation**							
OHCA, n (%)	104	(68.9)	0	(0.0)	104	(100.0)	**0.001**
IHCA, n (%)	47	(31.1)	47	(100.0)	0	(0.0)	
Shockable rhythm, n (%)	73	(49.0)	33	(71.7)	40	(38.8)	**0.001**
Non-shockable rhythm, n (%)	76	(51.0)	13	(28.3)	63	(61.2)	
No-flow time, min (median, IQR)	0	(0–7)					
Low-flow time, min (median, IQR)	5	(0–9)	0	(0–0)	6	(1–9)	0.062
Epinephrine, mg (median, IQR)	2	(1–6)	1	(1–6)	3	(1–7)	0.228
ROSC, min (median, IQR)	15	(10–27)	10	(5–22)	15	(10–29)	**0.017**
TTM, n (%)	69	(45.7)	6	(12.8)	63	(60.6)	**0.001**
** Coronary status**							
Coronary angiography, n (%)	84	(55.6)	29	(61.7)	55	(52.9)	0.466
Time from CS onset to coronary angiography, min (median, IQR)	135	(86–291)	262	(73–628)	121	(86–169)	**0.029**
Patients with culprit lesion, n (%)	85	(56.3)	30	(63.8)	55	(52.9)	0.211
- LMT	6	(3.9)	3	(6.4)	3	(2.9)	0.402
- LAD	39	(25.8)	13	(27.7)	26	(25.0)	
- RCA	16	(10.6)	4	(8.5)	12	(11.5)	
- RCX	20	(13.3)	8	(17.0)	12	(11.5)	
- RIM	2	(1.3)	1	(2.1)	1	(0.9)	
- CABG	1	(0.7)	0	(0)	1	(0.9)	
** Respiratory status**							
Mechanical ventilation, n (%)	127	(87.6)	29	(67.4)	98	(96.1)	**0.001**
Duration of mechanical ventilation, days, (mean, (IQR))	3	(1–7)	2	(1–2)	5	(2–9)	**0.001**
PaO_2_/FiO_2_ ratio on admission, (median, (IQR))	194	(118–319)	197	(114–302)	192	(120–331)	0.668
PaO_2_ on admission, mmHg (median, (IQR))	108	(77–187)	92	(75–174)	112	(79–201)	0.240
PaCO_2_ on admission, mmHg (median, (IQR))	45	(36–52)	39	(34–47)	46	(38–54)	**0.018**
** Cardiovascular support during ICU**							
Dosis norepinephrine on admission, µg/kg/min (median, IQR)	0.2	(0.1–0.6)	0.2	(0.0–0.8)	0.1	(0.1–0.3)	0.837
Dobutamine, µg/kg/min (median, IQR)	4.0	(3.0–6.0)	4.0	(3.0–6-0)	4.0	(2.5–6.0)	0.850
Time to administration of dobutamine, days (median, IQR)	3	(0–6)	5	(0–7)	2	(0–5)	0.316
Levosimendan, n (%)	33	(22)	11	(23.4)	22	(21.2)	0.679
Time to administration of levosimendan, days (median, IQR)	2	(1–3)	1	(1–2)	2	(2–3)	**0.007**
Mechanical circulatory assist device, n (%)	22	(14.6)	9	(19.1)	13	(12.5)	0.284
- va-ECMO	20	(13.2)	8	(17.0)	12	(11.5)	0.359
- Impella	4	(3.6)	2	(4.3)	2	(1.9)	0.410
- Both devices	2	(1.3)	1	(2.1)	1	(0.9)	0.280
Duration of mechanical circulatory assist device, min (median, IQR)	3	(1–5)	1	(1–4)	3	(2–5)	0.118
** Dialysis support during ICU**							
CRRT, n (%)	43	(28.5)	13	(27.7)	29	(27.9)	0.746
Duration of CRRT, h (median, IQR)	42	(16–83)	16	(10–27)	70	(31–108)	**0.008**

CABG, coronary artery bypass graft; CRP, C-reactive protein; CRRT, continuous renal replacement therapy; CS, cardiogenic shock; va-ECMO, veno-arterial extracorporeal membrane oxygenation; ICU, intensive care unit; IHCA, in-hospital cardiac arrest; IQR, interquartile range; LAD, left anterior descending; LMT, left main trunk; OHCA, out-of-hospital cardiac arrest; TAPSE, tricuspid annular plane systolic excursion; RCA, right coronary artery; RCX, ramus circumflexus; RIM; ramus interventricularis; ROSC, return of spontaneous circulation; TTM, targeted temperature management; level of significance *p* < 0.05. Bold type indicates statistical significance.

**Table 4:** Uni- and multivariable Cox regression analyses within the entire study cohort.

Variables	Univariate			Multivariate		
	HR	95% CI	*p*-Value	HR	95% CI	*p*-Value
Age	1.013	0.998–1.027	0.091	1.004	0.987–1.021	0.624
BMI	1.036	0.990–1.084	0.127	1.027	0.967–1.091	0.380
Heart rate > 110 bpm	1.936	1.207–3.105	**0.006**	1.367	0.797–2.347	0.256
Respiratory rate ≥ 22/min	1.634	1.056–2.529	**0.028**	1.070	0.639–1.792	0.796
WBC (10^6^/mL)	1.011	0.979–1.045	0.509	1.023	0.983–1.065	0.256
Platelets (10^6^/mL)	0.998	0.996–1.000	0.109	0.996	0.993–1.000	**0.040**
cTNI (µg/L)	1.002	1.000–1.003	**0.008**	1.001	1.000–1.003	**0.045**
Creatinine (mg/dL)	1.189	1.027–1.377	**0.021**	1.127	0.949–1.338	0.173
IHCA	1.480	0.977–2.241	0.064	1.794	1.053–3.056	**0.031**

BMI, body mass index; cTNI, cardiac troponin I; IHCA, in-hospital cardiac arrest; WBC, white blood cell count. Level of significance *p* < 0.05.

**Table 5:** Multivariable Cox regression analyses stratified by AMI.

Variables	Non-AMI			AMI		
	HR	95% CI	*p*-Value	HR	95% CI	*p*-Value
Age	1.016	0.984–1.049	0.322	0.997	0.976–1.019	0.807
BMI	0.993	0.905–1.090	0.879	1.060	0.969–1.159	0.203
Heart rate > 110 bpm	0.428	0.150–1.226	0.114	1.959	0.985–3.895	0.055
Respiratory rate ≥ 22/min	1.891	0.782–4.574	0.157	0.864	0.442–1.691	0.670
WBC (10^6^/mL)	1.100	1.023–1.183	**0.010**	0.976	0.922–1.034	0.415
Platelets (10^6^/mL)	0.991	0.985–0.998	**0.014**	0.998	0.994–1.002	0.332
cTNI (µg/L)	1.033	0.977–1.092	0.253	1.002	1.000–1.003	0.070
Creatinine (mg/dL)	1.119	0.735–1.704	0.600	1.151	0.945–1.401	0.161
IHCA	2.480	0.725–8.489	0.148	2.477	1.258–4.879	**0.009**

BMI, body mass index; cTNI, cardiac troponin I; IHCA, in-hospital cardiac arrest; WBC, white blood cell count. Level of significance *p* < 0.05.

## Data Availability

Not applicable.
